# Anxiety and Depressive Symptoms in the New Life With COVID-19: A Comparative Cross-Sectional Study in Japan Rugby Top League Players

**DOI:** 10.3389/ijph.2021.1604380

**Published:** 2022-01-18

**Authors:** Yasutaka Ojio, Asami Matsunaga, Shin Kawamura, Masanori Horiguchi, Goro Yoshitani, Kensuke Hatakeyama, Rei Amemiya, Ayako Kanie, Chiyo Fujii

**Affiliations:** ^1^ Department of Community Mental Health and Law, National Institute of Mental Health, National Center of Neurology and Psychiatry, Tokyo, Japan; ^2^ Japan Rugby Players’ Association, Tokyo, Japan; ^3^ Faculty of Health and Sports Sciences, University of Tsukuba, Tsukuba, Japan; ^4^ National Center for Cognitive Behavioral Therapy and Research, National Center of Neurology and Psychiatry, Tokyo, Japan

**Keywords:** mental health, COVID-19, rugby players, elite athlete, depression, anxiety, psychological distress, cluster-analysis

## Abstract

**Objectives:** The primary objective is to compare the prevalence of mental health problems, including psychological distress, anxiety and depressive symptoms in Japan Rugby Top League players in the new life with COVID-19 with those evaluated before COVID-19.

**Methods:** An observational comparative web-based cross-sectional study was employed for Japan Rugby Top League players. We compared the data from 220 Japanese and 7 foreign players during the new life with COVID-19 with the data from before COVID-19, which was obtained from 233 Japanese and 18 foreign players. We measured anxiety and depression symptoms with the validated Kessler-6, which has been widely used in clinical and research settings among different populations. To investigate the distribution of K6 score and whether there are discrete clusters or not, we conducted the two-step cluster analysis.

**Results:** In the new life with COVID-19, 15.0% of players reported mild symptoms, which was significantly lower than the 32.3% of players before COVID-19. The prevalence of moderate and severe symptoms was 6.7 and 3.5%, respectively, in the group during the new life with the COVID-19, and 4.8 and 5.2% in the pre-COVID-19 group, with no significant difference. A two-step cluster analysis supported the existence of these two qualitatively different clusters in both groups.

**Conclusions:** With the spread of new lifestyles related to COVID-19, some rugby players may have improved mental health status due to changes in their daily living environment. Such environmental adjustments alone may not have been sufficient to change the mental health status of others. Rugby players or their teams may require mental health professionals and systems that ensure rest, adjust the environment, and sustainably provide more professional care.

## Highlights

• The study provides insight into the impact of the huge social and environmental changes resulting from the COVID-19 pandemic on the mental health of the elite athletes in the Japan Rugby Top League. Such an impact is of increasing clinical and research interest worldwide.
• Few epidemiological studies have been conducted to examine the mental health status of elite athletes in Asian countries, including Japan, and the current findings are gained from a different sample than previous studies in this research field.
• A particular strength of the current study is the use of Kessler-6, which is already globally validated and widely used in clinical and research settings in different populations, which could allow comparison with other populations or conditions.
• A limitation of the study is that we conducted a cross-sectional study rather than a prospective cohort study.
• A further limitation is possible bias due to mental health-related stigma and pandemic-related stressor self-reporting experiences.

## Introduction

Research and practice on mental health among elite athletes is growing rapidly. The prevalence of mental health symptoms and illnesses in elite sport is comparable to the general population. According to a recent review by Reardon et al. (2019), [1] the prevalence ranges from 5 to 35% for anxiety and depression in elite athletes reported in previous prospective studies, which also applies to male rugby players in United Kingdom [2] and Japan. [3] The multiple factors of the mental health problems include general factors (e.g., stressful life events and inadequate social support) and athlete-specific factors (e.g., physical injury, poor performance, competition for selection, career transition). In addition, contact sports athletes may exhibit mental health symptoms and associated suicidal behavior due to biological causes such as chronic traumatic encephalopathy [4]. The usual definition of an elite athlete is an athlete who competes at a national or international level, including athletes in the domestic league [5]. International sports organizations, including the International Olympic Committee (IOC), and experts from sports sciences have stated that mental health research is required for elite athletes [1, 6–11]. The essential first step for developing care is understanding the actual mental health status.

The COVID-19 pandemic has changed the daily lives of people worldwide, and athletes have also been affected by the spread of the infection. For example, lockdown in the place of residence, postponement of the Tokyo 2020 Olympic and Paralympic Games and other competitions, and restricted use of training centers have been implemented as measures to prevent the spread of infection. These changes and the associated adaptation to behavioral restrictions may have caused demoralization due to the accumulation of multiple reasons [12, 13]. The social conditions may also have increased mental health problems, including depression and anxiety [14–16]. Gouttebarge et al. compared the mental health status measured by PHQ-9 of European soccer league players before COVID-19 and during the COVID-19 emergency period [16]. In the COVID-19 emergency period, both male and female soccer players had more clinical mental health symptoms than before the COVID-19 infection. A year since the spread of COVID-19, at the time when the current research was conducted, people in Japan were beginning to adapt to the social changes in their lives due to COVID-19. Office work had been changed to working remotely from home, eliminating the responsibility of going to the office and increasing self-organization of time. This change was also relevant to the athletes in this study, as Japan Rugby Top League is a corporate sport, and many athletes are employed by a company. Most media events attended by athletes had also changed from in person to online. Although different from life before COVID-19, due to measures such as wearing masks to prevent infection, in Japan training and practice had resumed, and competitions were gradually being held.

Regarding the mental health status of elite athletes, comparisons between the pre-COVID-19 period and the initial emergency phase and lockdown period have been reported [16–18]. However, to the best of our knowledge, no studies have compared the pre-COVID-19 period with life after changes in the social environment due to COVID-19. The purpose of this study is to compare the prevalence of mental health problems, including psychological distress, anxiety and depressive symptoms in Japan Rugby Top League players in the new life with COVID-19 with those evaluated before COVID-19.

## Methods

### Study Design

This study is an observational comparative cross-sectional study. Our report is in accordance with the Strengthening the Reporting of Observational Studies in Epidemiology (STROBE) guidelines [19, 20] for observational cross-sectional studies.

### Participants and Recruitment

We recruited the participants from a total of 612 rugby players (565 Japanese and 47 foreign players) registered with the Japan Rugby Players’ Association. The participants were all aged 18 years and over and belonged to the Japan Rugby Top League. The current survey was available in both Japanese and English. Exclusion criteria did not apply. The current study employed a web-based cross-sectional design. This survey URL was distributed to all the players who were members of the Japan Rugby Players’ Association through representatives of each team. The participants were invited to complete the anonymous survey submitted only by those who voluntarily agreed. The completion time of the survey was estimated to be less than 10 min. The participants were informed *via* the cover page on the web about the survey process, including the purpose of the study, data collection procedures, and the consequences of participating or not participating in the study. The participants were reminded of any missing items before progressing to the next page, resulting in no missing outcome data. We granted the participants individual, one-time access to complete the survey *via* a tablet or computer using IP address filtering. The cross-sectional data were collected shortly before the start of the off-season from December 2020 to February 2021.

### Measurements

This survey was conducted as part of a collaborative project with researchers, mental health professionals, and the Japan Rugby Players’ Association to develop a mental health care system for Japanese elite athletes, including Japan Rugby Top League players [21]. The current study design and the content of this web-survey were developed by an interdisciplinary team consisting of rugby players, psychiatrists, clinical psychologists, psychiatric social workers and public health nurses.

### Background Information/Demographics

The survey of background information and demographic items included age, country of birth, educational attainment, marital status, a child living in the household, residential status, experience on the national team, and status of play during the last season.

### Main Outcome Measures

We measured anxiety and depression symptoms with the Japanese version of a globally widely accepted screening tool, the Kessler-6 (K6) [22, 23]. The K6 is a six-item self-report scale that evaluates the frequency of experiencing general psychological distress, such as nervousness, tiredness, hopelessness, and restlessness. The scores are categorized to indicate the respondents’ mental health problems over the past 30 days. Responses to items are made on a 5-point scale. Community samples have demonstrated the validity in screening mood and anxiety disorders and have been identified as a powerful psychometric test in the Japanese population [24]. Scores range from 0 to 24, with higher scores indicating more severe mental illness. Previous studies have suggested that psychological distress levels can be divided into four groups: no distress, mild, moderate, and severe distress, from 0 to 4, 5 to 10, 10 to 12, and 13, respectively [24, 25]. K6 has been translated into Japanese, and studies to establish screening performance and optimal cut-off points in Japan have shown that the optimal cut-off point for K6 is 4/5, and sensitivity is 100%, with moderate-to-high specificity [25]. The K6 has been widely used in clinical and research settings among different populations, including general populations, patients, and athletes worldwide. Thus, it offers normative data for comparison.

### Pre-COVID-19 Comparison Group

The comparison group consisted of male rugby players who belonged to the Japan Rugby Top League, as did those currently surveyed during the new life with COVID-19 [3]. The players in the comparison group fulfilled the same inclusion criteria as the current study group. The data from the comparison group was collected before the COVID-19 pandemic, from December 2019 to January 2020. Out of the total of 600 players registered with the Japan Rugby Players’ Association, 251 participants then gave their consent (response rate: 41.8%). The results of the previous sample have already been published [3].

### Patient and Public Involvement

No patients were involved in this study because the purpose was to assess the mental health status of Japan Rugby Top League players. Each participant received a summary of their findings from the Japan Rugby Players’ Association.

### Statistical Methods

The percentage of players with specific demographic characteristics and experience of certain life events was calculated according to anxiety and depression symptoms within the validated cut-off values of K6. The 30-days prevalence of anxiety and depressive symptoms (expressed as a percentage) was calculated for each level of severity as the proportion of the number of participants with the condition relative to the total number of participants. The differences between groups in the 30-days mental health status were determined with Fisher’s exact test. To determine which categories differed between the two groups, we performed residual analysis with the Bonferroni correction. In addition, we used two-step cluster analysis to investigate the distribution of K6 score and whether there were discrete clusters or not. To perform Fisher’s exact test and the residual analysis, we used the free software R. All other analyses were conducted with Stata version 16 (StataCorp LLC, College Station, TX, United States). All tests were two-sided, and *p*-values were compared with the significance level of *α* = 0.05.

### Ethical Considerations

All investigators received instruction on research ethics. This study was approved and facilitated by the Research Ethics Committee at the National Center of Neurology and Psychiatry (approval number: A2020-015 and A2020-058).

## Results

Among 565 Japanese players, 220 players agreed and completed the survey. Out of the total of 47 foreign players, 7 participants gave their consent. Overall, 227 of the 612 players agreed to complete the survey (response rate: 37.1%). The response rate of this survey was not lower than other mental health surveys in Japan. The pre-COVID-19 comparison group consisted of 233 Japanese and 18 foreign players. Overall, the new life with COVID-19 and pre-COVID-19 groups were similar regarding descriptive variables (Table 1).

**TABLE 1:** Demographic characteristics of the study participants before-COVID-19 (n = 251) and in the new life with COVID-19 (n = 227). Mental fitness survey for rugby players, Japan, 2019–2021.

	Before COVID-19 n = 251, % (n)	Life with COVID-19 n = 227, % (n)
Age at survey		
≤19	0.4 (1)	0 (0)
20–24	19.9 (50)	26.9 (61)
25–29	51.8 (130)	45.4 (103)
30–34	24.7 (62)	22.9 (52)
35 ≥	3.2 (8)	4.9 (11)
Educational attainments		
High school or vocational college	2.8 (7)	3.1 (7)
Four-year college or university	95.6 (240)	96.0 (218)
Postgraduate college (or more)	1.6 (4)	0.9 (2)
Marital status		
Married	47.0 (118)	47.1 (107)
Never married	51.0 (128)	52.0 (118)
Divorced or widowed	2.0 (5)	0.9 (2)
Child living in household	30.3 (76)	24.7 (56)
Residential Status		
Living alone	19.5 (49)	23.8 (54)
With family or partner	50.2 (126)	47.1 (107)
Dormitory	30.3 (76)	29.1 (66)
Experience on national team	21.9 (55)	19.8 (45)
Status of play last season		
As a starting member	32.3 (81)	35.7 (81)
As a reserve member	31.9 (80)	27.8 (63)
No play	35.9 (90)	36.6 (83)

Table 2 presents the prevalence and score of psychological distress and anxiety and depressive symptoms (per severity level) for the pre-COVID-19 and new life with COVID-19 groups. 32.3% of the players in the pre-COVID-19 group and 15.0% of the players in the new life with COVID-19 reported mild symptoms. The prevalence of moderate and severe symptoms was 10.0% (4.8 and 5.2%, respectively) in the pre-COVID-19 group and 10.2% (6.7 and 3.5%, respectively) in the new life with COVID-19. Fisher’s exact test showed a significant difference in distribution between the two groups (x^2^ = 21.6, df = 3, *p* < 0.001). The residual analysis showed that in the new life with COVID-19 group, the frequency of normal status was significantly higher than the expected frequency (z = 3.943, adjusted *p* = 0.001), and the frequency of mild symptoms was significantly lower than the expected frequency (z = −4.417, adjusted *p* < 0.001). Bonferroni correction was used to adjust the *p*-value.

**TABLE 2:** Prevalence of anxiety and depressive symptoms for each level of severity before COVID-19 (n = 251) and in the new life with COVID-19 Group (n = 227). Mental fitness survey for rugby players, Japan, 2019–2021.

	Before COVID-19 (n = 251)	Life with COVID-19 (n = 227)
Anxiety and depression symptoms (% (n))		
Normal (score 0–4)	57.8 (145)	74.9 (170)
Mild (score 5–10)	32.2 (81)	15.0 (34)
Moderate (score 10–12)	4.8 (12)	6.7 (15)
Severe (score 13–24)	5.2 (13)	3.5 (8)

The two-step cluster analysis showed that both groups were divided into two clusters. The mean scores for K6 were 1.4 ± 1.6 and 10.1 ± 3.8 in the new life with COVID-19 groups, and 1.9 ± 1.6 and 8.5 ± 3.4 in the pre-COVID-19 groups, respectively.

## Discussion

In the current cross-sectional comparative study, we have demonstrated that the proportion of Japan Rugby Top League male players with psychological distress had decreased over the preceding year of COVID-19 social change. We have also shown that the prevalence of anxiety and depressive symptoms in elite athletes was consistent between the pre-COVID-19 period and the period 1 year after the COVID-19 infection emergency period.

### Mild Depression and Anxiety Symptoms

The current results show that in the new life with COVID-19, when some stability had been achieved in the economy and daily life in Japan, the prevalence of psychological distress in players had decreased compared with the prevalence prior to COVID-19. Given that clinical mental health symptoms, including anxiety and depression, increased with the initial spread of the infection, when there were severe restrictions on behavior such as lockdown, [16] most players in the current survey appear to have adapted and experienced recovery as the social environment changed after the initial state of emergency. Previous findings [17, 18] showed that the high-level athletes received the impacts on a dichotomized mental health by the COVID-19; they experienced a period of overwhelmingly stress and followed by a return to baseline well-being during the lockdown. Elite athletes are known to be an at-risk but resilient population, resourceful in achieving positive adjustments [26]. While the players experienced social changes due to COVID-19, their mental health status may have been positively impacted by the guarantee of their basic livelihood and salary by their team and company. Paid jobs other than competition and training were moved online. The resulting increase in self-organization of time that the players could use to recover from physical and mental fatigue may have positively affected mental health. Their mental health status may also have improved with the recommencement of the schedule of the Japan Rugby Top League. In some rugby players, as in the general population, there may be a subpopulation in which environmental adjustment works effectively.

### Moderate or Severe Depression and Anxiety Symptoms

We also found no difference in the proportion of players with clinical mental health symptoms, including anxiety and depression, during social and environmental changes due to COVID-19. However, we should note the interpretation of this result. Since the time-trend comparison was at the cross-sectional group level rather than the individual level using longitudinal data, players with clinical mental health symptoms are not necessarily the same individuals in both groups. While the number of players with mild symptoms decreased due to environmental and lifestyle changes, moderate to severe cases remained unchanged. The current results might indicate that significant environmental and lifestyle changes alone could not modify or improve the poor mental health status of some players with moderate to severe mental health symptoms. In other words, moderate to severe mental health status in rugby players or athletes might be related to something other than environmental problems, such as lack of rest time. This finding may be useful information for clinical mental health care. According to a supporting module by the IOC mental health working group from a psychiatric treatment perspective, [27] this subpopulation might require additional care, including brief psychotherapy, [28] and in some cases medication, in addition to environmental adjustments.

### Findings From Two-Step Cluster Analysis

The two-step cluster analysis results also showed two discrete clusters in both of the two periods. The results suggest that the Japan Rugby Top League players may be composed of two qualitatively different groups. They may consist of a “standard” cluster without or with mild psychological distress resulting from a normal response to environmental factors, and a “pathological” cluster with mental health problems. This information could be shared among athletes, coaches and healthcare professionals working in the sports community, and they might consider implementing prevention, early detection, and support and recovery measures [29]. The findings indicate that rugby players or their teams need mental health professionals and systems that ensure rest, adjust the environment, and sustainably provide more professional care. Universal mental health care by non-clinician (e.g., athlete welfare staff, coaches/paraprofessionals) can be incorporated into a team or organization. Such non-clinical approaches, i.e., adequate rest, mental health status measurement, or consideration of additional mental health support, might help maintain optimal health [29, 30]. Additionally, for the pathological group, more specialized treatment might be required, such as psychotherapy and/or medication. In Australia, a research and practice collaboration between Orygen Youth Health and the Australian Institute of Sport has been implemented with such a specific mental health care system for elite athletes [31].

### Strengths and Limitations

We acknowledge the strengths of this study and several limitations that should be taken into consideration. One of the strengths is that it is one of the few studies investigating the impact of huge social and environmental changes on an elite athlete population. While globally rising clinical and research interests drive the move to promote and support elite athletes’ mental health, only a small number of fundamental epidemiological studies have investigated Japanese athletes’ mental health. Regarding the limitations of this study, first, we conducted a cross-sectional study rather than a prospective cohort study. A rigorous prospective study based on a longitudinal design with a one-year follow-up period, including the COVID-19 emergency period, might provide insight into time-based trends. Second, the participants in this study were all male rugby players, considering the feasibility of the study. While the response rate to this survey is comparable to other mental health surveys, [32] it is not sufficient to accurately estimate Japanese elite athletes’ overall trend. People who do not respond to or are unwilling to engage in such mental health surveys may have mental health-related stigma and more severe symptoms [33]. In addition, an international comparative survey is required because there may be significant cross-cultural differences between different cultures in mental health symptoms and illnesses and the implementation of preventative measures for infectious diseases. Third, the variables related to risk factors of mental health problems rather than COVID-19 life changes in the athletes examined in this study might not be sufficient. Third, since the variables included in the data at the two time points treated in this study were the same, we were able to compare them. However, the variables related to risk factors of mental health problems in the athletes examined in this study might not be sufficient. Based on the results of previous research [18], in future research we need to expand the scope of adjustment risk variables, including injuries, relocation, or being on tour/long periods away from home.

### Conclusion

Overall, Japanese male rugby players’ mental health status as elite athletes in the new life with COVID-19 was better than before COVID-19. However, the proportion of players who had clinical mental health symptoms did not changed during this period. In the new lifestyle associated with COVID-19, the mental health status of some rugby players might have improved due to changes in the social environment, such as increased self-organization of time, based on guaranteed security of livelihood and salary. In contrast, such environmental adjustments alone may not have changed the mental health status of other players. Additional two-step cluster analysis might clarify the existence of two qualitatively different groups. The mental health status of a “standard” group might result from normal responses to the surrounding environment. The mental health status of others might be more “pathological” and require professional care in addition to environmental adjustment. Mental health professionals and systems that provide continuous mental health care with multiple options might have to be incorporated in teams or organizations.

## Data Availability

Data cannot be shared publicly because there exist ethical restrictions. Publishing data sets is not covered by the informed consent provided by the study participants, which was approved by the ethics committee of the Research Ethics Committee at the National Center of Neurology and Psychiatry. In the approval, the use, including viewing and analysis of the data, was limited to the researchers who applied. The data are not owned by a third party. Non-author contact information for the body imposing the restrictions upon the data, to which data access requests can be sent, is the Research Ethics Committee of the National Center of Neurology and Psychiatry (rinri-jimu@ncnp.go.jp).

## References

[1] Reardon CL Hainline B Aron CM Baron D Baum AL Bindra A Mental Health in Elite Athletes: International Olympic Committee Consensus Statement (2019). Br J Sports Med (2019) 53:667–99. 10.1136/bjsports-2019-100715 31097450

[2] Nicholls AR Madigan DJ Fairs LRW Bailey R. Mental Health and Psychological Well-Being Among Professional rugby League Players from the UK. BMJ Open Sport Exerc Med (2020) 6:e000711. 10.1136/bmjsem-2019-000711 PMC7047489 32153985

[3] Ojio Y Matsunaga A Hatakeyama K Kawamura S Horiguchi M Yoshitani G Anxiety and Depression Symptoms and Suicidal Ideation in Japan Rugby Top League Players. Ijerph (2021) 18(3):1205. 10.3390/ijerph18031205 33572911 PMC7908153

[4] Costanza A Radomska M Zenga F Amerio A Aguglia A Serafini G Severe Suicidality in Athletes with Chronic Traumatic Encephalopathy: A Case Series and Overview on Putative Ethiopathogenetic Mechanisms. Ijerph (2021) 18(3):876. 10.3390/ijerph18030876 33498520 PMC7908343

[5] Swann C Moran A Piggott D. Defining Elite Athletes: Issues in the Study of Expert Performance in Sport Psychology. Psychol Sport Exerc (2015) 16:3–14. 10.1016/j.psychsport.2014.07.004

[6] Moesch K Kenttä G Kleinert J Quignon-Fleuret C Cecil S Bertollo M. FEPSAC Position Statement: Mental Health Disorders in Elite Athletes and Models of Service Provision. Psychol Sport Exerc (2018) 38:61–71. 10.1016/j.psychsport.2018.05.013

[7] Schinke RJ Stambulova NB Si G Moore Z. International Society of Sport Psychology Position Stand: Athletes’ Mental Health, Performance, and Development. Int J Sport Exerc Psychol (2017) 16:622–39. 10.1080/1612197x.2017.1295557

[8] Breslin G Smith A Donohue B Donnelly P Shannon S Haughey TJ International Consensus Statement on the Psychosocial and Policy-Related Approaches to Mental Health Awareness Programmes in Sport. BMJ Open Sport Exerc Med (2019) 5:e000585. 10.1136/bmjsem-2019-000585 PMC6797268 31673406

[9] Gorczynski P Gibson K Thelwell R Papathomas A Harwood C Kinnafick F. The BASES Expert Statement on Mental Health Literacy in Elite Sport. Sport Exerc Sci (2019) 59:6–7.

[10] Henriksen K Schinke R Moesch K McCann S Parham WD Larsen CH Consensus Statement on Improving the Mental Health of High Performance Athletes. Int J Sport Exerc Psychol (2019) 18:553–560. 10.1080/1612197x.2019.1570473

[11] Van Slingerland KJ Durand-Bush N Bradley L Goldfield G Archambault R Smith D Canadian Centre for Mental Health and Sport (CCMHS) Position Statement: Principles of Mental Health in Competitive and High-Performance Sport. Clin J Sport Med (2019) 29:173–80. 10.1097/jsm.0000000000000665 31033609

[12] Costanza A Di Marco S Burroni M Corasaniti F Santinon P Prelati M Meaning in Life and Demoralization: a Mental-Health reading Perspective of Suicidality in the Time of COVID-19. Acta Biomed (2020) 91(4):e2020163. 10.23750/abm.v91i4.10515 33525223 PMC7927501

[13] Costanza A Baertschi M Richard-Lepouriel H Weber K Berardelli I Pompili M Demoralization and its Relationship with Depression and Hopelessness in Suicidal Patients Attending an Emergency Department. Ijerph (2020) 17(7):2232. 10.3390/ijerph17072232 PMC7177663 32225017

[14] Mehrsafar AH Gazerani P Moghadam Zadeh A Jaenes Sánchez JC. Addressing Potential Impact of COVID-19 Pandemic on Physical and Mental Health of Elite Athletes. Brain Behav Immun (2020) 87:147–8. 10.1016/j.bbi.2020.05.011 32387513 PMC7201218

[15] Reardon CL Bindra A Blauwet C Budgett R Campriani N Currie A Mental Health Management of Elite Athletes during COVID-19: A Narrative Review and Recommendations. Br J Sports Med (2021) 55(11):608–615. 10.1136/bjsports-2020-102884 32967853

[16] Gouttebarge V Ahmad I Mountjoy M Rice S Kerkhoffs G. Anxiety and Depressive Symptoms during the Covid-19 Emergency Period: A Comparative Cross-Sectional Study in Professional Football. Clin J Sport Med (2020). 10.1097/jsm.0000000000000886. 32941374

[17] Balcombe L De Leo D. Psychological Screening and Tracking of Athletes and Digital Mental Health Solutions in a Hybrid Model of Care: Mini Review. JMIR Form Res (2020) 4(12):e22755. 10.2196/22755 33271497 PMC7746225

[18] Simons C Martin LA Balcombe L Dunn PK Clark RA. Mental Health Impact on At-Risk High-Level Athletes during COVID-19 Lockdown: A Pre-, during and post-lockdown Longitudinal Cohort Study of Adjustment Disorder. J Sci Med Sport (2021) 24(4):329–31. 10.1016/j.jsams.2020.12.012 33402273 PMC8837901

[19] von Elm E Altman DG Egger M Pocock SJ Gøtzsche PC Vandenbroucke JP. The Strengthening the Reporting of Observational Studies in Epidemiology (STROBE) Statement: Guidelines for Reporting Observational Studies. Ann Intern Med (2007) 147(8):573–7. 10.7326/0003-4819-147-8-200710160-00010 17938396

[20] Vandenbroucke JP von Elm E Altman DG Gøtzsche PC Mulrow CD Pocock SJ Strengthening the Reporting of Observational Studies in Epidemiology (STROBE): Explanation and Elaboration. Ann Intern Med (2007) 147(8):W163–94. 10.7326/0003-4819-147-8-200710160-00010-w1 17938389

[21] Ojio Y Matsunaga A Hatakeyama K Kawamura S Horiguchi M Baron D Developing a Japanese Version of the Baron Depression Screener for Athletes Among Male Professional rugby Players. Ijerph (2020) 17(15):E5533. 10.3390/ijerph17155533 32751819 PMC7432524

[22] Prochaska JJ Sung H-Y Max W Shi Y Ong M. Validity Study of the K6 Scale as a Measure of Moderate Mental Distress Based on Mental Health Treatment Need and Utilization. Int J Methods Psychiatr Res (2012) 21:88–97. 10.1002/mpr.1349 22351472 PMC3370145

[23] Furukawa TA Kawakami N Saitoh M Ono Y Nakane Y Nakamura Y The Performance of the Japanese Version of the K6 and K10 in the World Mental Health Survey Japan. Int J Methods Psychiatr Res (2008) 17:152–8. 10.1002/mpr.257 18763695 PMC6878390

[24] Nishi D Imamura K Watanabe K Ishikawa H Tachimori H Takeshima T Psychological Distress with and without a History of Depression: Results from the World Mental Health Japan 2nd Survey (WMHJ2). J Affective Disord (2020) 265:545–51. 10.1016/j.jad.2019.11.089 31759666

[25] Sakurai K Nishi A Kondo K Yanagida K Kawakami N. Screening Performance of K6/K10 and Other Screening Instruments for Mood and Anxiety Disorders in Japan. Psychiatry Clin Neurosci (2011) 65(5):434–41. 10.1111/j.1440-1819.2011.02236.x 21851452

[26] Gupta S McCarthy PJ. Sporting Resilience during COVID-19: What Is the Nature of This Adversity and How Are Competitive Elite Athletes Adapting? Front Psychol (2021) 12:611261. 10.3389/fpsyg.2021.611261 33746833 PMC7966721

[27] Gouttebarge V Bindra A Blauwet C Campriani N Currie A Engebretsen L International Olympic Committee (IOC) Sport Mental Health Assessment Tool 1 (SMHAT-1) and Sport Mental Health Recognition Tool 1 (SMHRT-1): Towards Better Support of Athletes' Mental Health. Br J Sports Med (2021) 55(1):30–7. 10.1136/bjsports-2020-102411 32948518 PMC7788187

[28] Stillman MA Glick ID McDuff D Reardon CL Hitchcock ME Fitch VM Psychotherapy for Mental Health Symptoms and Disorders in Elite Athletes: A Narrative Review. Br J Sports Med (2019) 53:767–71. 10.1136/bjsports-2019-100654 30944086

[29] Purcell R Gwyther K Rice SM. Mental Health in Elite Athletes: Increased Awareness Requires an Early Intervention Framework to Respond to Athlete Needs. Sports Med Open (2019) 5:46–8. 10.1186/s40798-019-0220-1 31781988 PMC6883009

[30] Gouttebarge V Bindra A Blauwet C Campriani N Currie A Engebretsen L International Olympic Committee (IOC) Sport Mental Health Assessment Tool 1 (SMHAT-1) and Sport Mental Health Recognition Tool 1 (SMHRT-1): Towards Better Support of Athletes’ Mental Health. Br J Sports Med (2021) 55:30–7. 10.1136/bjsports-2020-102411 32948518 PMC7788187

[31] Rice S Butterworth M Clements M Josifovski D Arnold S Schwab C Development and Implementation of the National Mental Health Referral Network for Elite Athletes: A Case Study of the Australian Institute of Sport. Case Stud Sport Exerc Psychol (2020) 4:S1–27. 10.1123/cssep.2019-0016

[32] Kawakami N Yasuma N Watanabe K Ishikawa H Tachimori H Takeshima T Association of Response Rate and Prevalence Estimates of Common Mental Disorders across 129 Areas in a Nationally Representative Survey of Adults in Japan. Soc Psychiatry Psychiatr Epidemiol (2020) 55:1373–82. 10.1007/s00127-020-01847-3 32047970

[33] Perales Perez F Baffour B. Respondent Mental Health, Mental Disorders and Survey Interview Outcomes. Surv Res Methods (2018) 12(2):161–76. 10.18148/srm/2018.v12i2.7225

